# Association between disability, social support and depressive symptoms in Chinese older adults: A national study

**DOI:** 10.3389/fpubh.2022.980465

**Published:** 2022-08-19

**Authors:** Gang Tian, Rui Li, Yiran Cui, Tong Zhou, Yan Shi, Wenyan Yang, Yulan Ma, Jingliang Shuai, Yan Yan

**Affiliations:** Department of Epidemiology and Medical Statistics, XiangYa School of Public Health, Central South University, Changsha, China

**Keywords:** depressive symptoms, social support, moderating effect, elderly people, disability

## Abstract

**Objective:**

Disability and social support can impact depressive symptoms of the elderly. Yet, studies infrequently discuss the moderating role of social support when evaluating the association between disability and depressive symptoms. The purpose of this study was to explore the association between disability, social support, and depressive symptoms among the Chinese elderly, and further examine the moderating effect of social support.

**Materials and methods:**

Using the 2018 Chinese Longitudinal Healthy Longevity Survey (CLHLS) data set, we finally selected 9,231 Chinese elderly after screening. The Center for Epidemiologic Studies Depression Scale (CESD-10) was used to evaluate depressive symptoms in the elderly. Disability was measured by basic activities of daily living (B-ADL) and instrumental activities of daily living (I-ADL). Social support included contact with family and friends, sick care, and money received, measured by five self-reported questions. We used multiple linear regression and moderating model to explore the association between disability, social support, and depressive symptoms.

**Results:**

A total of 9,231 patients were included in this study, and approximately 26.75% of the elderly had depressive symptoms. Study found that depressive symptoms were associated with social support (β_*B*−*ADL*_ = −0.108, 95% CI: −0.168– −0.047; β_*I*−*ADL*_ = −0.098, 95% CI: −0.156– −0.039), β_*B*−*ADL*_ (β = 0.296, 95% CI: 0.248−0.343) and I-ADL (β = 0.174, 95% CI: 0.152–0.195). Moreover, the result also showed that social support moderated the effects of B-ADL ($\beta_{B-ADL^{*}socialsupport}$ = 0.034, 95% CI: 0.014–0.053, F = 11.57, *p* = 0.001) and I-ADL ($\beta_{I-ADL^{*}socialsupport}$ = 0.025, 95% CI: 0.017–0.033) on depressive symptoms.

**Conclusions:**

The study suggests that disability and social support can affect depressive symptoms, and social support moderates the effect of disability on depressive symptoms. Therefore, taking effective measures to reduce the elderly disability rate of disability and increase their social support are necessary condition for realizing mental health.

## Introduction

Most countries in the world, including China, are experiencing serious aging problems (1). According to the seventh National Census in 2021, the number of Chinese over 65 years old has reached 190 million, accounting for 13.50% of the total population, and the proportion of the population aged 65 and above rose by 4.63 percentage points (2). From 2005 to 2017, the life expectancy of Chinese residents showed an upward trend. However, the accompanying health problems have seriously affected the physical and mental health of the elderly (3–5). The World Health Organization (WHO) expects that the disease burden of depression will top all other diseases by 2030 (6). In China (2019), depression is one of the top ten causes of disability-adjusted life years (DALYs) (7). Studies have shown that depressive symptoms are a highly prevalent disease in elderly people (8). Beyond personal suffering and family disruption, depression worsens the outcomes of many medical disorders and promotes disability (9, 10).

Functional ability refers to the ability of individuals to participate in social activities according to their intentions and life preferences. It is usually measured by basic activities of daily living (B-ADL) and instrumental activities of daily living (I-ADL) (11). At present, the current situation of disability in the elderly is not optimistic. A study in European countries shows that the I-ADL disability rate of the elderly over 65 years old was 23.8% (12), while the B-ADL and I-ADL disability rates of the elderly over 60 years old in China are 23.8 and 35.4%, respectively (13). Since the elderly with disability usually need help from family or caregivers, their self-choice ability decreases and their social networks shrink (14–16). The elderly often experience low self-control ability and a strong sense of loneliness or meaninglessness, which are the risk factors for increased depressive symptoms in the elderly (16–19). Therefore, the disability of the old adults seriously affects the quality of life of the elderly and brings a burden to the family and society.

With the increase of age, it is inevitable to suffer the disability. Therefore, how to reduce the health problems brought by the disability to the elderly, such as depressive symptoms, was particularly important. It is worth noting that social support, especially from family and friends, is likely to be one factor that can play a key role in against depression for older adults (20). For example, previous studies have shown that older people with limited social support are vulnerable to psychological problems, such as depression and anxiety (21, 22). Although previous studies have indicated the association between disability, social support, and depressive symptoms (20, 23, 24), few studies have discussed the potential role of social support in assessing the association between disability and depressive symptoms. A study indicated that social networks with sufficient scale, quality, and interaction frequency will give the elderly a feeling of being valued and loved (25). Especially when the elderly are unable to integrate into social groups due to restricted movement, the support from family and friends can alleviate psychological pressure, give the elderly a sense of security and relief, and eventually improve life satisfaction (26–29). Therefore, social support, especially from family and friends, may moderate the effect of disability on depressive symptoms in the elderly.

From what has been discussed above, we can see that there is some association between disability, social support, and depressive symptoms. Therefore, one of the purposes of this study is to explore the association between disability, social support, and depressive symptoms among the Chinese elderly, and further examine the moderating effects of social support. To provide a theoretical basis for the prevention and improvement of depressive symptoms in the elderly.

## Methods and measurements

### Data source and sample

The data in this study came from the Chinese Longitudinal Healthy Longevity Survey (CLHLS 2017-2018). CLHLS is a follow-up survey of the elderly in China organized by the Center for Healthy Aging and Development Studies (CHADS) of Peking University, which was firstly conducted in 1998. Based on the baseline survey, CLHLS conducted another seven surveys in 2000, 2002, 2005, 2008-2009, 2011–2012, 2014, and 2017–2018. The main subjects of this survey are elderly people over 65 years old in 23 Chinese provinces. Considering that the sample data should be representative and reliable, the sampling design of CLHLS adopted a multi-stage disproportionate and targeted random sampling method (30). In addition, more details about the CLHLS survey have been described in other studies (31). In the latest survey, CLHLS collected information on their depressive symptoms, the activity of daily living, and social support, as well as demographic, behavioral, and health-related information. Inclusion criteria in this study were age ≥ 65 (15773). Additionally, the cases with missing values and outliers from the main variables (n = 6,542) were further excluded, detail are shown in the Supplementary Figure 1. Therefore, the final sample was 9,231 elderly respondents, which were included in the analysis.

### Measurements

#### Depressive symptoms

The Center for Epidemiologic Studies Depression Scale (CESD-10) was used to evaluate depressive symptoms in the elderly. The CESD-10 consisted of 10 items using a 4-point Likert scale. For the two positive questions, “I was happy” and “I felt hopeful about the future,” answers were reversely coded before summation. We then coded all answers from 0 to 3 as “rarely” to “most of the time,” respectively. The total range of CESD-10 scores in this study was 0–30, with higher scores indicating greater severity of depressive symptoms. A person is considered to have depressive symptoms if he/she scored no <10 on the CESD-10 (32). Previous studies have confirmed the reliability and effectiveness of CESD-10 in measuring depressive symptoms in older adults (32).

#### Disability

Disability was measured by B-ADL and I-ADL. B-ADL was measured with the following six subscales (1) Bathing; (2) Dressing; (3) Toileting; (4) Indoor moving; (5) Continence of defecation; (6) Eating. I-ADL was rated with eight questions (1) Can you visit your neighbors by yourself? (2) Can you go shopping by yourself? (3) Can you cook a meal by yourself when necessary? (4) Can you wash clothes by yourself when necessary? (5) Can you walk a kilometer at a time by yourself? (6) Can you lift a weight of 5 kg, such as a heavy bag of groceries? (7) Can you continuously squat and stand up three times? (8) Can you take public transportation by yourself? Each item was scored from 0 (complete independence) to 2 (complete dependence). The more scores the respondents obtained, the higher B-ADL and I-ADL dependence would be. In addition, a question measured the overall activity ability of the elderly, “for the last 6 months, were you limited in activities because of a health problem?” and coded all answers from 1 to 3 as “yes, strongly limited” to “not limited,” respectively.

#### Social support

Social support from family and friends included contact with family and friends, sick care, and money received (whether participants received money from children). Three questions measured contact with family and friends, “contact1: to whom do you usually talk most frequently in daily life?” “contact2: to whom do you talk first when you need to share something of your thoughts?” and “contact3: who do you ask first for help when you have problems or difficulties?” Sick care is measured in one sentence, “who takes care of you when you are sick?” Answer options included spouse, son, daughter, daughter-in-law, son-in-law, grandchildren, other relatives, friends/neighbors, social workers, housekeeper, or nobody. If the person was the spouse, the item scored 3. If the person was a child, friend, or relative, the item scored 2. If the person was a social worker or housekeeper, the item scored 1. If the option was nobody, the item scored 0. Finally, one question measured money received, “Do you receive money from the children,” if an answer was yes, then the answer was coded as 1, otherwise, it was coded as 0 (33). These five items were summed up with a score ranging from 0 to 13 and higher scores denoting more extensive social support.

#### Covariate

Control variables include demographic variables (age, sex, education, and rural residence), behavioral variables (marital status, living pattern, physical exercise, smoking, and drinking), and subjective relative poverty. Physical exercise is measured by “Do you often exercise now? (refers to purposeful fitness activities, such as walking, playing ball, running, square dance, Tai Chi, etc.).” Subjective relative poverty is measured in one sentence, “how do you rate your economic status compared with other local people?” We then coded all answers from 1 to 5 as “very rich” to “very poor,” respectively.

### Statistical analysis

In this study, depression symptoms score was the response variable, disability was the predictor and the social support moderator variable. Descriptive analysis was used to describe the general characteristics of the study population, one-way analysis of variance (ANOVA), *t*-test, and χ^2^ test were performed to compare depression symptoms score between different groups. Moreover, to prove whether social support plays a moderating role between disability and depressive symptoms, we used moderating effect model to verify the impact of interaction items on depressive symptoms, and the mathematical formula is as follows:


$$\begin{array}{l} Y=\beta_{0}+\beta_{disability}X+\beta_{social\;support}M\\ \;+\beta_{disability*social\;support}XM+\varepsilon \end{array}$$

All statistical tests were two-sided, and *P* < 0.05 was considered statistically significant. Statistical analyses were performed using SPSS 26.0 and R 4.0.0. We used SPSS to describe the basic information of the study population and establish the moderating effect model. R performs data visualization and correlation test.

## Results

### Characteristics of the study sample

Participants' characteristics are shown in Table 1. In the present study, more than 50% of the older adults were female and most of the participants lived in rural regions. Of the 9231 participants, 2469 (26.75%) developing depressive symptoms. After case weighted, the mean age was 72.35 years (standard deviation = 6.55), 70.60% were married and living with a spouse, 14.40% living alone or in an institution, and 27.60% of the participants were uneducated. Additionally, 80.10% of the participants currently do not smoke, 81.50% do not drink, and 57.50% rarely took part in physical exercise. Depressive symptoms participants are more likely to be women, living in rural, less educated, never married, living in pension institutions, subjective relative poverty, and rarely participate in physical exercise.

**Table 1:** Describe the characteristics of depressive symptoms in Chinese adults aged 65 years and older.

**Variables**	***n***	Percent*	Depression symptoms score*	*P*-value*	**Depression**		*P*-value*
					**No**	**Yes**	
**Age group, years**							
65–79	3,784	84.10%	6.72 ± 4.34	<0.001	2,923	861	<0.001
80–99	4,288	15.80%	7.62 ± 4.51		3,032	1,256
≥100	1,159	0.01%	7.63 ± 5.49		807	352
**Sex**							
Men	4,260	47.40%	6.39 ± 4.11	<0.001	3,316	944	<0.001
Women	4,971	52.60%	7.29 ± 4.56		3,446	1,525	
**Education level, years**							
0	4,026	27.60%	7.90 ± 4.52	<0.001	2,694	1,332	<0.001
1–6	3,213	42.00%	6.69 ± 4.15		2,461	752
≥7	1,992	30.40%	6.17 ± 4.38		1,607	385
**Residence**							
City	2,338	22.80%	6.66 ± 4.66	<0.001	1,831	507	<0.001
Town	3,036	29.80%	7.03 ± 4.48		2,152	884	
Rural	3,857	47.40%	6.86 ± 4.16		2,779	1,078	
**Marital status**							
Married and living with spouse	4,195	70.60%	6.49 ± 4.20	<0.001	3,275	920	<0.001
Separated	172	2.40%	7.03 ± 4.30		123	49
Divorced	32	0.50%	7.50 ± 3.85		24	8
Widowed	4,769	25.80%	7.80 ± 4.69		3,307	1,462
Never married	63	0.70%	8.89 ± 4.77		33	30
**Living pattern**							
With household member(s)	7,415	85.60%	6.64 ± 4.25	<0.001	5,585	1,830	<0.001
Alone	1,505	12.80%	8.06 ± .79		984	521
In an institution	311	1.60%	9.09 ± 5.24		193	118
**Subjective poverty**							
Very rich	261	2.50%	5.29 ± 3.89	<0.001	225	36	<0.001
Rich	1,614	16.00%	5.50 ± 3.87		1,377	237
General level	6,446	71.40%	6.77 ± 4.12		4,719	1,727
Poor	802	8.90%	9.90 ± 5.09		397	405
Very poor	108	1.10%	11.82 ± 6.79		44	64
**Smoking**							
Yes	1,490	19.90%	6.49 ± 4.04	<0.001	1,168	322	<0.001
No	7,741	80.10%	6.96 ± 4.45		5,594	2,147
**Drinking**							
Yes	1,398	18.50%	5.92 ± 4.09	<0.001	1,124	274	<0.001
No	7,833	81.50%	7.08 ± 4.41		5,638	2,195
**Physical exercise**							
Yes, often	3,245	42.50%	6.02 ± 4.00	<0.001	2,684	561	<0.001
No, rarely	5,986	57.50%	7.49 ± 4.54		4,078	1,908

*Case weighted results.

### Association between disability, social support and depressive symptoms

**Figure 1:**
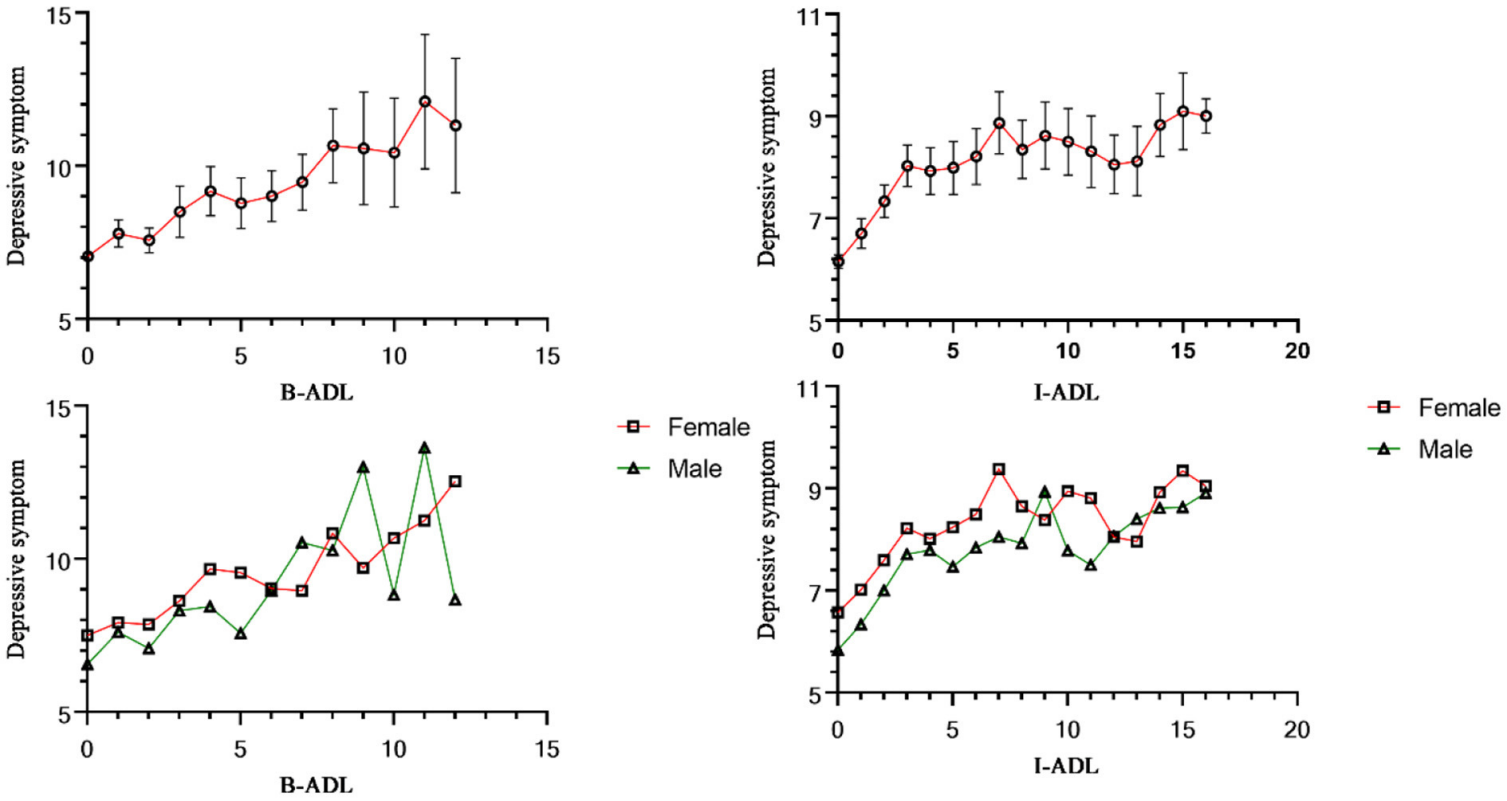
Association between disability and depressive symptoms in the elderly.

Of the 9,231 individuals, 2,867 (31.06%) activities of daily living were limited due to health problems, of which 771 were strongly limited. Figure 1 showed the association between disability and depressive symptoms. B-ADL (*r* = 0.170, *P* < 0.001) and I-ADL (*r* = 0.224, *P* < 0.001) were significantly correlated with depressive symptoms. In addition, if men and women were limited by the same activities of daily living, the depression symptoms score of women is higher than men (*P* < 0.05), detailed data are shown in the Supplementary Table 1. However, when the limitation of activities of daily living was higher than a certain threshold, this difference will no longer be obvious. Specifically, when B-ADL was not <6 and I-ADL was not <9.

**Figure 2:**
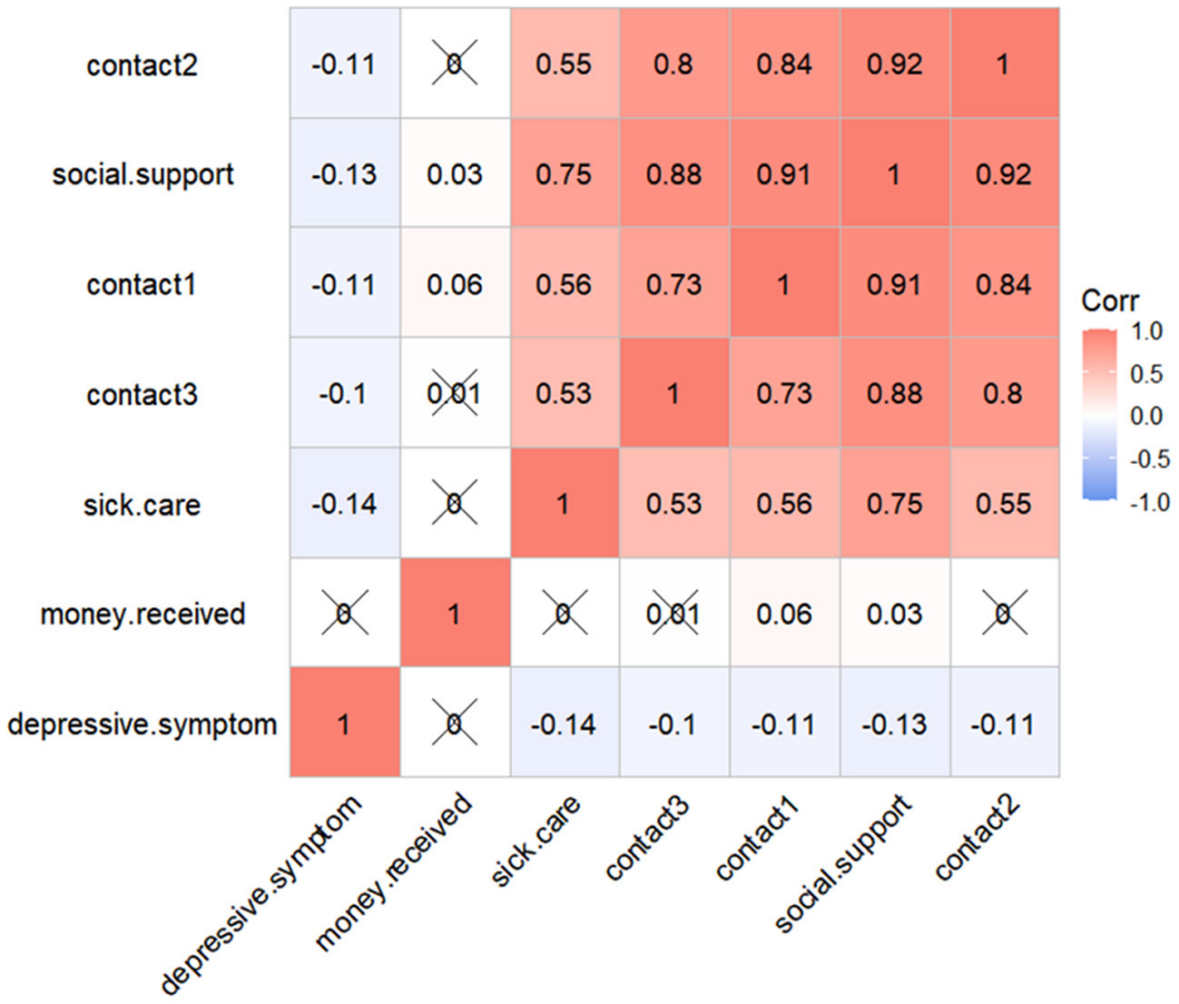
Correlation coefficient of social support and depressive symptoms. ^×^*P* > 0.05.

**Figure 3:**
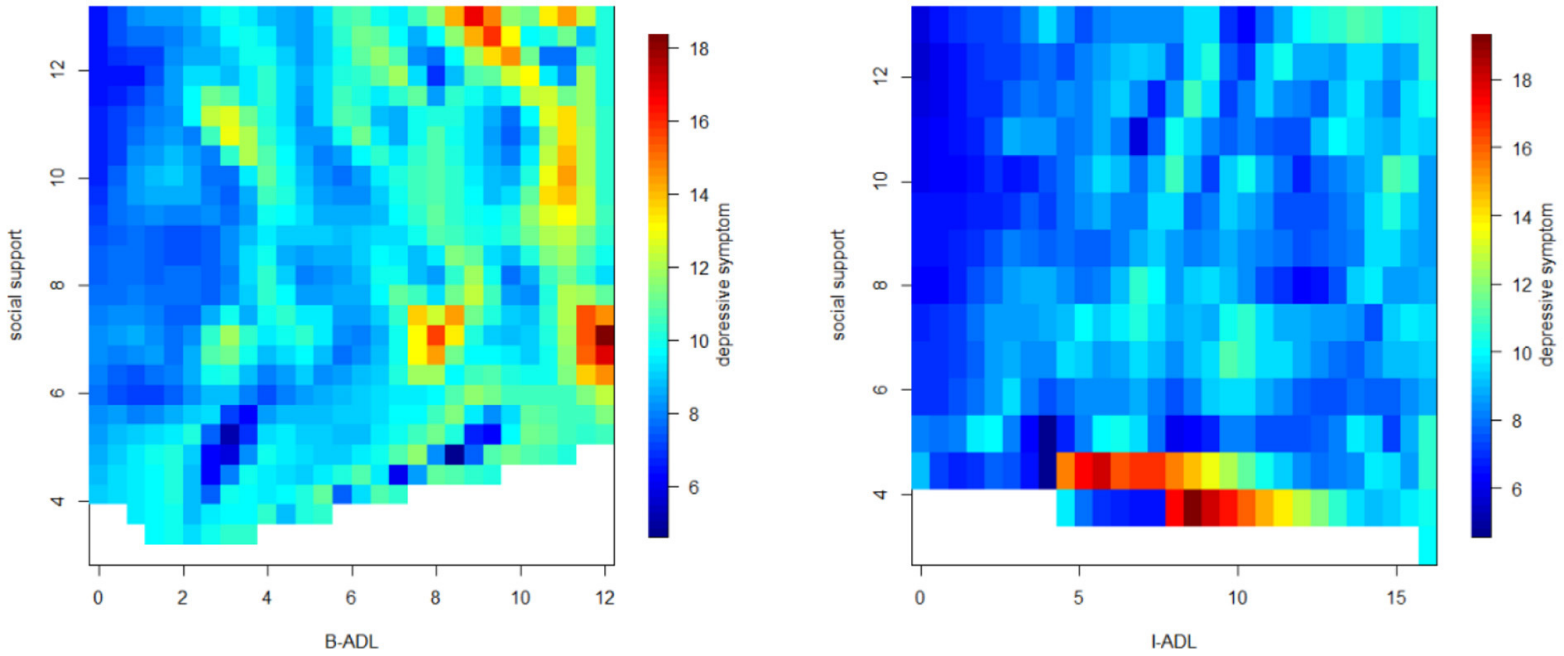
Association between disability, social support, and depressive symptoms in the elderly.

Figure 2 indicated that social support (*r* = −0.13, *P* < 0.001) was significantly correlated with depressive symptoms. Specifically, sick care (*r* = −0.14, *P* < 0.001), contact1 (*r* = −0.11, *P* < 0.001), contact2 (*r* = −0.11, *P* < 0.001), and contact3 (*r* = −0.10, *P* < 0.001) were negatively correlated with depressive symptoms. Finally, Figure 3 showed the association between disability, social support, and depressive symptoms. The elderly with less social support and higher disability were more likely to be depressed.

### The moderating role of social support in the effect of disability on depressive symptoms

**Figure 4:**
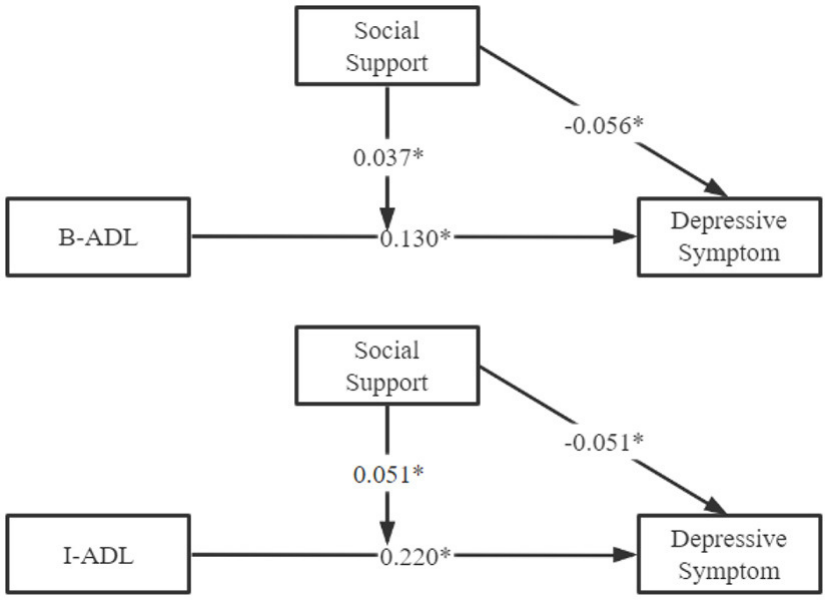
Model of moderation by social support. *P < 0.05.

The moderating effect of social support between disability and depressive symptoms was shown in Tables 2, 3. After controlling for potential confounders, the results showed that the effects of social support (β_*B*−*ADL*_ = −0.108, 95% CI: −0.168– −0.047; β_*I*−*ADL*_ = −0.098, 95% CI: −0.156– −0.039), B-ADL (β = 0.296, 95% CI: 0.248–0.343) and I-ADL (β = 0.174, 95% CI: 0.152–0.195) on depression were statistically significant. Moreover, social support moderated the effects of B-ADL (β_*B*−ADL*socialsupport_ = 0.034, 95% CI: 0.014–0.053, *F* = 11.57, *p* = 0.001) and I-ADL (β_*I*−ADL*socialsupport_ = 0.018, 95% CI: 0.010–0.025, *F* = 23.93, *p* < 0.001) on depressive symptoms. Additionally, to better explain the regulatory role of social support, we visualized the association between social support, disability, and depressive symptoms, as shown in Figure 4.

**Table 2:** Moderation analysis of social support for the association between B-ADL and depressive symptoms.

**Variable**	**Standardized** **coefficients**	**Coefficients**	**SE**	***t-*value**	***P-*value**	**95% CI**	
						**Lower**	**Upper**
**Model 1 (unadjusted) (*****R**^**2**^* **=** **0.04** ***F*** **=** **127.134** ***p*** **<** **0.001)**							
***R**^**2**^* **change due to the moderator** **=** **0.002 (*****F*** **=** **20.99**, ***p*** **<** **0.001)**							
B-ADL	0.170	0.387	0.023	16.523	<0.001	0.342	0.432
Social support	−0.096	−0.184	0.020	−9.17	<0.001	−0.223	−0.145
B-ADL*social support	0.054	0.050	0.011	4.582	<0.001	0.028	0.072
**Model 2 (adjusted) (*****R**^**2**^* **=** **0.130 F** **=** **84.96** ***p*** **<** **0.001)**							
***R**^**2**^* **change due to the moderator** **=** **0.001 (*****F*** **=** **11.57**, ***p*** **=** **0.001)**							
B-ADL	0.130	0.296	0.024	12.075	<0.001	0.248	0.343
Social support	−0.056	−0.108	0.031	−3.527	<0.001	−0.168	−0.047
B-ADL*social support	0.037	0.034	0.010	3.188	0.001	0.014	0.053

Model 2 adjusted for age, gender, educational level, subjective poverty, marital status, living pattern, residence, smoking, drinking, and physical exercise. Set residence, living pattern, and marital status as dummy variables. SE: standard error; CI: confidence interval; disability *social support: the interaction effect between disability and social support.

**Table 3:** Moderation analysis of social support for the association between I-ADL and depressive symptoms.

**Variable**	**Standardized coefficients**	**Coefficients**	**SE**	***t*-value**	***p-*value**	**95% CI**	
						**Lower**	**Upper**
**Model 3 (unadjusted) (*****R**^**2**^* **=** **0.057** ***F*** **=** **186.49** ***p*** **<** **0.001)**							
**R**^**2**^ **change due to the moderator** **=** **0.004 (*****F*** **=** **43.97**, ***p*** **<** **0.001)**							
I-ADL	0.224	0.177	0.008	22.087	<0.001	0.161	0.192
Social support	−0.053	−0.102	0.021	−4.908	<0.001	−0.143	−0.060
I-ADL*social support	0.071	0.025	0.004	6.631	<0.001	0.017	0.033
**Model 4 (adjusted) (*****R**^**2**^* **=** **0.143** ***F*** **=** **94.89** ***p*** **<** **0.001)**							
***R**^**2**^* **change due to the moderator** **=** **0.003(*****F*** **=** **23.93**, ***p*** **<** **0.001)**							
I-ADL	0.220	0.174	0.011	16.407	<0.001	0.152	0.195
Social support	−0.051	−0.098	0.030	−3.231	0.001	−0.156	−0.039
I-ADL*social support	0.051	0.018	0.004	4.836	<0.001	0.010	0.025

Model 4 adjusted for age, gender, educational level, subjective poverty, marital status, living pattern, residence, smoking, drinking, and physical exercise. Set residence, living pattern, and marital status as dummy variables. SE: standard error; CI: confidence interval; disability *social support: the interaction effect between disability and social support.

## Discussion

The purpose of the study was to explore the association between disability, social support, and depressive symptoms, and further examine whether social support could moderate the association between disability and depressive symptoms among the Chinese elderly. After adjusting for potential confounding factors, the results showed that disability and social support significantly affected depressive symptoms in the elderly. Meanwhile, social support played a potentially moderating effect between disability and depressive symptoms of the elderly. Therefore, the findings of this study supported that social support was an effective way to promote positive and healthy aging, especially among older adults with disability.

In this study, we found that approximately 26.75% of the Chinese elderly had depressive symptoms. This was similar to previous research, which showed that the prevalence of depressive symptoms was 25.55% among older Chinese (34). In addition, the current research shows that about 31.06% of the elderly have limited activities of daily living due to health reasons. This was also consistent with previous research, which reported that the B-ADL and I-ADL disability rates of the elderly over 60 years old in China were 23.8 and 35.4%, respectively (13). With the increase of age, the activities of daily living of the elderly gradually decreased. A study, based on six provinces in China, reported that the incidence of disability among 23,803 participants aged 60 and over was 12% during a 2-year follow-up (35). Although the overall disability rate had a slight downward trend with the development of social economy and the improvement of medical levels, the disability rate of rural elderly people still showed an upward trend (36). Thus, the current situation of disability and depressive symptoms in the Chinese elderly was not optimistic.

This study's results suggested that activities of daily living negatively correlated with depressive symptoms in the Chinese elderly. In addition, results also showed that social support, especially from spouses and children, significantly affected the depressive symptoms of the elderly. Those were in agreement with the previous research (37–40). Elderly with disabilities increased the likelihood of comorbidity, early mortality, and mental health problems, which increased the obstacles for the elderly to achieve a high quality of life (41, 42). Nevertheless, family support, especially emotional support, was an important guarantee to maintaining the mental health of the elderly (43). Based on the above, we tried to reveal the potential association between disability, social support, and depressive symptoms of the elderly in China. The current study found that social support can moderate the effect of disability on depressive symptoms. This finding may be explained by the theory of psychological elasticity, which refers to demonstrating positive psychological results in the face of adversity (44). When the activities of daily living of the elderly was limited, the effective mobilization of psychological and social resources can increase the psychological elasticity of the elderly, which was fundamental to achieving good outcomes (45). It could be argued that social support can alleviate psychological stress and reduce the feelings of helplessness brought by disability to the elderly, thereby decreasing depressive symptoms. Therefore, social support was a crucial factor in preventing depression in the elderly and buffering the impact of disability on their mental health.

The strength of this study is that study used data from a national survey, the population is representative. In addition, the study explored the potential moderating role of social support between disability and depressive symptoms in the elderly. There is no denying that there are still many shortcomings in this study. First of all, this research design was a cross-sectional study, all questions were self-reported, which cannot determine the causal association between variables. Furthermore, social support as a protective factor against depressive symptoms of the elderly, can improve the quality of life of the elderly and have a positive impact on actively coping with aging. Therefore, it may be more instructive to explore the impact of social support from specific groups, such as spouses, sons, daughters, and grandchildren, on depressive symptoms of the elderly in the future.

In conclusion, this study found that disability and social support can affect depressive symptoms among older adults. In addition, social support moderates the effect of disability on depressive symptoms. Now the prevalence of depression in the elderly is increasing year by year, which brings a serious burden to the family and society. Taking effective measures to reduce the elderly disability rate and increase their social support is a necessary condition for realizing mental health. Therefore, future studies should explore diversified pension models to meet the needs of the elderly. On the one hand, institutions could integrate eldercare services with medical care to meet the needs of the elderly, especially for the elderly living alone. On the other hand, communities can be linked by kinship, to realize the life concept of two generations living together, and increase the communication between the elderly and family members.

## Data availability statement

The original contributions presented in the study are included in the article/Supplementary material, further inquiries can be directed to the corresponding author.

## Author contributions

RL designed the study question, performed the statistical analyses, and wrote the first draft and revision. GT performed the statistical analyses and critical revision. YC, TZ, YS, WY, YM, and JS critical revision. YY was responsible for the overall supervision of the study design and revised the manuscript. All authors read and approved the final manuscript.

## Conflict of interest

The authors declare that the research was conducted in the absence of any commercial or financial relationships that could be construed as a potential conflict of interest.

## Publisher's note

All claims expressed in this article are solely those of the authors and do not necessarily represent those of their affiliated organizations, or those of the publisher, the editors and the reviewers. Any product that may be evaluated in this article, or claim that may be made by its manufacturer, is not guaranteed or endorsed by the publisher.

## References

[1] Beard JR Officer A de Carvalho IA Sadana R Pot AM Michel J-P. The World report on ageing and health: a policy framework for healthy ageing. Lancet. (2016) 387:2145–54. 10.1016/S0140-6736(15)00516-4 26520231 PMC4848186

[2] Statistics NBo. Bulletin of China's Seventh National Census (No.5). (2021). Available online at: http://www.stats.gov.cn/tjsj/tjgb/rkpcgb/qgrkpcgb/202106/t20210628_1818824.html (accessed March 15, 2022).

[3] Fane M Weeraratna AT. How the ageing microenvironment influences tumour progression. Nat Rev Cancer. (2020) 20:89–106. 10.1038/s41568-019-0222-9 31836838 PMC7377404

[4] O'Rourke N. Mental health and aging in Israel: emerging and longstanding successes and challenges. Aging Ment Health. (2020) 24:523–4. 10.1080/13607863.2020.1711869 31920089

[5] Yin P Jin Q Liu Y Liu J Li J Zeng X. Burden of disease in the Chinese population from 2005 to 2017. Chinese Circ J. (2019) 34:1145–54. 10.3969/j.issn1000-3614.2019.12.00131474069

[6] Malhi GS Mann JJ. Depression. Lancet. (2018) 392:2299–312. 10.1016/S0140-6736(18)31948-2 30396512

[7] WHO. Top 10 Causes of DALY in China for Both Sexes Aged All Ages. (2019). Available online at: https://www.who.int/data/gho/data/themes/mortality-and-global-health-estimates/global-health-estimates-leading-causes-of-dalys (accessed March 15, 2022).

[8] Almeida OP. Prevention of depression in older age. Maturitas. (2014) 79:136–41. 10.1016/j.maturitas.2014.03.005 24713453

[9] Cui R. A systematic review of depression. Curr Neuropharmacol. (2015) 13:480. 10.2174/1570159X1304150831123535 26412067 PMC4790400

[10] Alexopoulos GS. Depression in the elderly. Lancet. (2005) 365:1961–70. 10.1016/S0140-6736(05)66665-2 15936426

[11] Beard JR Officer AM Cassels AK. The world report on ageing and health. Gerontologist. (2016) 56:S163–6. 10.1093/geront/gnw037 26994257

[12] Portela D Almada M Midão L Costa E. Instrumental activities of daily living (iADL) limitations in Europe: an assessment of SHARE data. Int J Environ Res Public Health. (2020) 17:7387. 10.3390/ijerph17207387 33050460 PMC7599802

[13] Qian J Wu K Luo H Cao P Ren X. Prevalence of loss of activities of daily living and influence factors in elderly population in China. Chin J Epidemiol. (2016) 37:1272–6. 10.3760/cma.j.issn.0254-6450.2016.09.018 27655577

[14] Zhang MW Chan S Wynne O Jeong S Hunter S Wilson A. Conceptualization of an evidence-based smartphone innovation for caregivers and persons living with dementia. Technol Health Care. (2016) 24:769–73. 10.3233/THC-161165 27129032

[15] Dunlop DD Hughes SL Manheim LM. Disability in activities of daily living: patterns of change and a hierarchy of disability. Am J Public Health. (1997) 87:378–83. 10.2105/AJPH.87.3.378 9096537 PMC1381008

[16] Burholt V Windle G Morgan DJ. A social model of loneliness: the roles of disability, social resources, and cognitive impairment. Gerontologist. (2017) 57:1020–30. 10.1093/geront/gnw125 27831482 PMC5881780

[17] HU Y Pengyue W Lihua L Cangmei F Xiuying F Jiaxin L. The effect of loneliness on the quality of life of the elderly in nursing home: chain mediation of depression and frailty. Modern Prev Med. (2020) 47:2801–5.

[18] Gong E Hua Y Yan LL. Psychological wellbeing and all-cause mortality in the oldest old in China: a longitudinal survey-based study. Lancet. (2016) 388:S22. 10.1016/S0140-6736(16)31949-3

[19] Li X Wang J Dong S Fu J Liu J. The influence of disabilities in activities of daily living on successful aging: the role of well-being and residence location. Front Public Health. (2019) 7:417. 10.3389/fpubh.2019.00417 32047732 PMC6997131

[20] Liu L Gou Z Zuo J. Social support mediates loneliness and depression in elderly people. J Health Psychol. (2016) 21:750–8. 10.1177/1359105314536941 24925547

[21] Liu LJ Guo Q. Life satisfaction in a sample of empty-nest elderly: a survey in the rural area of a mountainous county in China. Qual Life Res. (2008) 17:823–30. 10.1007/s11136-008-9370-1 18595006

[22] Cheng P Jin Y Sun H Tang Z Zhang C Chen Y. Disparities in prevalence and risk indicators of loneliness between rural empty nest and non-empty nest older adults in Chizhou, China. Geriatr Gerontol Int. (2015) 15:356–64. 10.1111/ggi.12277 24629147

[23] Barry LC Coman E Wakefield D Trestman RL Conwell Y Steffens DC. Functional disability, depression, and suicidal ideation in older prisoners. J Affect Disord. (2020) 266:366–73. 10.1016/j.jad.2020.01.156 32056900 PMC7103559

[24] Li A Wang D Lin S Chu M Huang S Lee CY. Depression and life satisfaction among middle-aged and older adults: mediation effect of functional disability. Front Psychol. (2021) 12:755220. 10.3389/fpsyg.2021.755220 34899497 PMC8656258

[25] Wang J Kong D Sun B Dong X. Health services utilization among Chinese American older adults: the role of social support. Innov Aging. (2018) 2:195–6. 10.1093/geroni/igy023.718 30033843 PMC6312757

[26] O'Donnell J Cardenas D Orazani N Evans A Reynolds KJ. The longitudinal effect of COVID-19 infections and lockdown on mental health and the protective effect of neighbourhood social relations. Soc Sci Med. (2022) 297:114821. 10.1016/j.socscimed.2022.114821 35219050 PMC8847081

[27] DuPertuis LL Aldwin CM Bosse R. Does the source of support matter for different health outcomes? findings from the normative aging study. J Aging Health. (2001) 13:494–510. 10.1177/089826430101300403 11917886

[28] Tsuji K Khan H. Exploring the relationship between social support and life satisfaction among rural elderly in Japan. Ageing Int. (2016) 41:1–13. 10.1007/s12126-016-9254-6

[29] Zhao X Zhang D Wu M Yang Y Xie H Li Y. Loneliness and depression symptoms among the elderly in nursing homes: a moderated mediation model of resilience and social support. Psychiatry Res. (2018) 268:143–51. 10.1016/j.psychres.2018.07.011 30025285

[30] Zheng Z. Twenty years' follow-up on elder people's health and quality of life. China Popul Dev Stud. (2020) 3:297–309. 10.1007/s42379-020-00045-7

[31] Zeng Y. Introduction to the Chinese Longitudinal Healthy Longevity Survey (CLHLS). Springer Netherlands (2008).

[32] Andresen EM Malmgren JA Carter WB Patrick DL. Screening for depression in well older adults: evaluation of a short form of the CES-D (Center for Epidemiologic Studies Depression Scale). Am J Prev Med. (1994) 10:77–84. 10.1016/S0749-3797(18)30622-6 8037935

[33] Yin S Yang Q Xiong J Li T Zhu X. Social support and the incidence of cognitive impairment among older adults in china: findings from the Chinese longitudinal healthy longevity survey study. Front Psychiatry. (2020) 11:254. 10.3389/fpsyt.2020.00254 32317993 PMC7154048

[34] Rong J Ge Y Meng N Xie T DIng H. Prevalence rate of depression in Chinese elderly from 2010 to 2019: a meta-analysis. Chin J Evid Based Med. (2020) 20:26–31. 10.7507/1672-2531.201908088

[35] Qi S Wang Z Wang L Wang H Zhang H Li Z. Incidence of activities of daily living disability and related factors in community-dwelling older adults in China. Chinese J Epidemiol. (2019) 40:272–6. 10.3760/cma.j.issn.0254-6450.2019.03.004 30884603

[36] Liu S Yin J Hong N. Epidemiological studies of activities daily living in the elderly. Chinese J Geriatric Care. (2022) 20:116–9+23. 10.3969/j.issn.1672-2671.2022.01.035

[37] Bozo O Toksabay NE Kurum O. Activities of daily living, depression, and social support among elderly Turkish people. J Psychol. (2009) 143:193–205. 10.3200/JRLP.143.2.193-206 19306681

[38] Unsar S Dindar I Kurt S. Activities of daily living, quality of life, social support and depression levels of elderly individuals in Turkish society. J Pak Med Assoc. (2015) 65:642–6. 26060163

[39] Yao R Guo M Ye H. The mediating effects of hope and loneliness on the relationship between social support and social well-being in the elderly. Acta Psychologica Sinica. (2018) 50:1151–8. 10.3724/SP.J.1041.2018.01151

[40] Chen LY Fang TJ Lin YC Hsieh HF. Exploring the mediating effects of cognitive function, social support, activities of daily living and depression in the relationship between age and frailty among community-dwelling elderly. Int J Environ Res Public Health. (2021) 18:12543. 10.3390/ijerph182312543 34886268 PMC8656521

[41] Millán-Calenti JC Tubío J Pita-Fernández S González-Abraldes I Lorenzo T Fernández-Arruty T. Prevalence of functional disability in activities of daily living (ADL), instrumental activities of daily living (IADL) and associated factors, as predictors of morbidity and mortality. Arch Gerontol Geriatr. (2010) 50:306–10. 10.1016/j.archger.2009.04.017 19520442

[42] Gayman MD Turner RJ Cui M. Physical limitations and depressive symptoms: exploring the nature of the association. J Gerontol B Psychol Sci Soc Sci. (2008) 63:S219–28. 10.1093/geronb/63.4.s219 18689771 PMC2844725

[43] Guo J Xu SL Chen L Zhu L. Impact of activities of daily living on depression in the elderly aged 60 and above in China. Chin J Epidemiol. (2022) 43:213–7. 10.3760/cma.j.cn112338-20210823-00667 35184487

[44] Masten A. Ordinary magic. resilience processes in development. Am Psychol. (2001) 56:227–38. 10.1037/0003-066X.56.3.227 11315249

[45] Ledesma. Conceptual frameworks and research models on resilience in leadership. Sage Open. (2014) 4. 10.1177/2158244014545464

