# Supplementary material for: Association between disability, social support and depressive symptoms in Chinese older adults: A national study
Source: Front Public Health. 2022 Aug 19;10:980465. doi: 10.3389/fpubh.2022.980465 (PMC9437525; doi:10.3389/fpubh.2022.980465)
Supplement: Supplementary file 1 [file Data_Sheet_1.docx]

**Supplementary Materials**


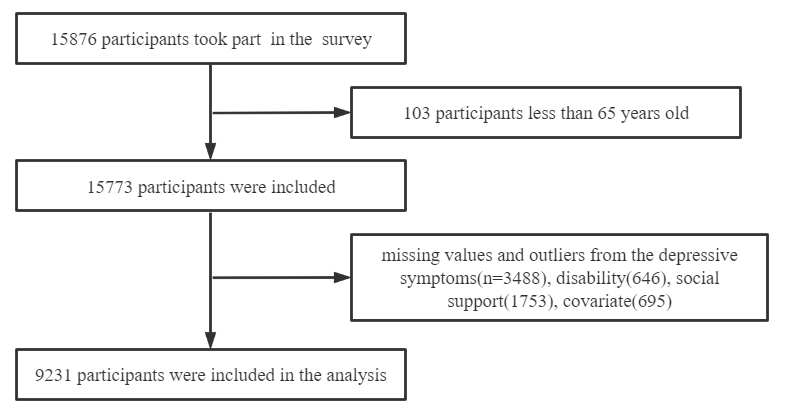


Supplementary Figure 1 Flow chart of study participants.

Supplementary Table1 Association between disability and depressive symptoms

|  |  | Female | | | Male | | | t | P |
| --- | --- | --- | --- | --- | --- | --- | --- | --- | --- |
|  |  | depression symptoms score | SE | n | depression symptoms score | SE | n |  |  |
| B-ADL | 0 | 7.5 | 0.07 | 3875 | 6.56 | 0.07 | 3608 | 568.84 | <0.05 |
|  | 1 | 7.92 | 0.31 | 235 | 7.61 | 0.34 | 161 | 9.44 | <0.05 |
|  | 2 | 7.86 | 0.28 | 288 | 7.08 | 0.31 | 172 | 27.97 | <0.05 |
|  | 3 | 8.63 | 0.63 | 72 | 8.31 | 0.55 | 51 | 2.92 | <0.05 |
|  | 4 | 9.67 | 0.55 | 86 | 8.45 | 0.61 | 60 | 12.57 | <0.05 |
|  | 5 | 9.55 | 0.56 | 76 | 7.57 | 0.62 | 49 | 18.61 | <0.05 |
|  | 6 | 9.04 | 0.52 | 90 | 8.96 | 0.74 | 54 | 0.76 | 0.4-0.5 |
|  | 7 | 8.96 | 0.52 | 83 | 10.54 | 0.95 | 39 | -11.83 | <0.05 |
|  | 8 | 10.84 | 0.73 | 64 | 10.28 | 1.16 | 32 | 2.88 | <0.05 |
|  | 9 | 9.71 | 0.92 | 31 | 13 | 2.68 | 11 | -6.01 | <0.05 |
|  | 10 | 10.68 | 0.91 | 38 | 8.83 | 4.59 | 6 | 2.34 | <0.05 |
|  | 11 | 11.25 | 1.40 | 20 | 13.64 | 2.13 | 11 | -3.78 | <0.05 |
|  | 12 | 12.54 | 1.24 | 13 | 8.67 | 2.62 | 6 | 4.45 | <0.05 |
| I-ADL | 0 | 6.57 | 0.11 | 1524 | 5.84 | 0.08 | 2046 | 231.04 | <0.05 |
|  | 1 | 7.02 | 0.21 | 416 | 6.34 | 0.20 | 360 | 45.67 | <0.05 |
|  | 2 | 7.59 | 0.21 | 372 | 7 | 0.24 | 285 | 33.61 | <0.05 |
|  | 3 | 8.22 | 0.26 | 290 | 7.71 | 0.35 | 183 | 18.20 | <0.05 |
|  | 4 | 8.02 | 0.30 | 238 | 7.79 | 0.37 | 166 | 6.85 | <0.05 |
|  | 5 | 8.24 | 0.33 | 207 | 7.47 | 0.45 | 100 | 17.05 | <0.05 |
|  | 6 | 8.49 | 0.38 | 167 | 7.84 | 0.42 | 125 | 13.81 | <0.05 |
|  | 7 | 9.38 | 0.41 | 144 | 8.05 | 0.47 | 88 | 22.47 | <0.05 |
|  | 8 | 8.65 | 0.39 | 140 | 7.93 | 0.42 | 99 | 13.51 | <0.05 |
|  | 9 | 8.38 | 0.39 | 109 | 8.94 | 0.58 | 85 | -8.03 | <0.05 |
|  | 10 | 8.95 | 0.45 | 125 | 7.78 | 0.46 | 78 | 17.78 | <0.05 |
|  | 11 | 8.81 | 0.48 | 109 | 7.5 | 0.54 | 68 | 16.89 | <0.05 |
|  | 12 | 8.05 | 0.40 | 135 | 8.06 | 0.44 | 88 | -0.18 | >0.50 |
|  | 13 | 7.96 | 0.45 | 103 | 8.4 | 0.54 | 58 | -5.50 | <0.05 |
|  | 14 | 8.93 | 0.39 | 160 | 8.62 | 0.56 | 81 | 5.04 | <0.05 |
|  | 15 | 9.35 | 0.50 | 116 | 8.63 | 0.59 | 63 | 8.62 | <0.05 |
|  | 16 | 9.05 | 0.20 | 616 | 8.91 | 0.32 | 287 | 7.98 | <0.05 |
